# Séroprévalence et facteurs associés au VIH et aux hépatites virales B et C dans la ville de Bafoussam au Cameroun

**DOI:** 10.11604/pamj.2015.20.156.4571

**Published:** 2015-02-19

**Authors:** Francois-Xavier Mbopi-Keou, Isabelle Vanessa Monthe Nkala, Ginette Claude Mireille Kalla, Georges Nguefack-Tsague, Hortense Gonsu Kamga, Michel Noubom, Côme Ebana Mvogo, Maurice Aurelien Sosso

**Affiliations:** 1 Faculty of Medicine & Biomedical Sciences, University of Yaoundé I, Yaounde, Cameroon; 2 Department of Laboratories and Blood Safety, Ministry of Public Health, Yaoundé, Cameroon; 3 Université des Montagnes, Bangangté, Cameroon; 4 University Teaching Hospital, Yaoundé-Cameroon; 5 University of Dschang, Dschang, Cameroon

**Keywords:** VIH, virus des hépatites B et C, séroprévalence, facteurs associés, Bafoussam, Cameroun, HIV, Hepatitis B and C virus, seroprevalence, associated factors, Bafoussam, Cameroon

## Abstract

**Introduction:**

L'objectif de ce travail était de déterminer la séroprévalence et les facteurs associés au VIH et aux hépatites virales B et C dans la ville de Bafoussam au Cameroun.

**Méthodes:**

Il s'agissait d'une étude descriptive et analytique réalisée de février 2012 à Juin 2012 dans la ville de Bafoussam au Cameroun. Pour cette étude, nous avons obtenu une clairance éthique.

**Résultats:**

Au total, 982 personnes ont été dépistées pour le VIH et les hépatites virales B et C. Les femmes représentaient 56,3% des personnes dépistées. La tranche d’âge la plus représentée était celle des 20 à 24 ans. L’âge médian était de 34,5 ans. Les prévalences du VIH, de l'AgHBs, et de l'Ac anti HCV étaient respectivement de 6,0%, 4,1%, et 0,4%. La prévalence du VIH était 2 fois plus élevée parmi les femmes que les hommes avec 8,1% contre 3,5% (p=0,01). Les prévalences les plus élevées ont été observées chez les personnes de 30 à 34 ans, 40 à 44 ans avec 15,0% et 11,5% (p=0,01), les personnes sans emploi avec 11,1% (p<0,001) et les personnes en union libre avec 17,9% (p=0,000). La prévalence du VIH n’était pas directement liée aux comportements et pratiques sexuels de la population de l’étude. On enregistrait une prévalence élevée de 29,3% chez les individus ayant déclaré avoir au moins une infection sexuellement transmissible (p=0,000).

**Conclusion:**

Il apparait urgent de mettre en place des stratégies de prévention contre le VIH, les hépatites virales et les facteurs associés au Cameroun.

## Introduction

La pandémie de l'infection par le Virus de l'Immunodéficience Humaine (VIH) est loin d’être contrôlée et ce nonobstant les grandes interventions menées à l’échelle planétaire. L′Afrique subsaharienne abrite plus de 67% de toutes les Personnes Vivant avec le VIH (PVVIH), soit 22,5 millions [1]. L′accès au traitement antirétroviral (TAR) en Afrique subsaharienne reste très faible en raison d′obstacles [2–4] tels que le nombre limité de médecins, la mesure de la charge virale limitée, la problématique de la disponibilité d′examens plus simples comme la mesure des lymphocytes T CD4 ou les examens de biochimie [1]. Devant ces difficultés, l′Organisation Mondiale de la Santé (OMS) a proposé une approche de prise en charge «allégée» pour favoriser l′accès aux antirétroviraux (ARV) à grande échelle dans les pays à ressources limitées [5]. Dès l'apparition des premiers cas de SIDA en 1985 au Cameroun [6], divers plans de lutte contre le VIH/SIDA ont été élaborés et mis en œuvre avec plus ou moins de succès de 1987 à nos jours [7]. Des progrès encourageants méritent d’être inscrits tels que l'accroissement important du nombre total des Centres de Traitement Agréés (CTA) et des Unités de Prise En charge du VIH/SIDA (UPEC), la gratuité des ARV depuis mai 2007, l'augmentation du nombre de personnes séropositives sous ARV [8]. Le Comité National de Lutte contre le SIDA (CNLS) rapporte en Décembre 2009 que sur 560 000 PVVIH, le Cameroun compterait près de 74.710 patients sous antirétroviraux (ARV), soit 39% des 153.185 PVVIH éligibles aux traitements dans l'ensemble du pays [9, 10]. Le VIH, avec sa virulence, ne constitue pas la seule menace sanitaire, il existe également d'autres virus dont l'expansion et l’évolution sont inquiétantes notamment le virus de l'hépatite B (VHB) et le virus de l'hépatite C (VHC) qui sont également des problèmes majeurs de santé publique. Pour le VHB, on estime que environ deux milliards de personnes dans le monde sont contaminées dont plus de 350 millions ont une atteinte hépatique chronique, et chaque année entre 500 000 et 700 000 personnes meurent de l'hépatite B [11]. Pour l'hépatite C, Quelques 130 à 170 millions de personnes en sont des porteurs chroniques et l'on estime à plus de 350 000 le nombre des décès annuels dus à des maladies du foie liées à l'hépatite C [11]. De plus, sur un plan thérapeutique, les deux virus hépatotropes nécessitent une prise en charge lourde, souvent mal tolérée conduisant le plus souvent des complications ce qui fait de la prévention le seul moyen de lutte efficace [11, 12]. L'ensemble de ces éléments plaident pour la nécessité d'un dépistage sérologique du VHB, du VHC et du VIH chez des personnes volontaires. Aussi, l'objectif de ce travail était de déterminer la séroprévalence et les facteurs associés au VIH et aux hépatites virales B et C dans la ville de Bafoussam au Cameroun.

## Méthodes

Nous avons mené une étude descriptive et analytique de février 2012 à Juin 2012 dans la ville de Bafoussam au Cameroun dans le cadre d'une campagne organisée par le Ministère de la Santé Publique avec les unités mobiles de dépistage [13, 14]. Le choix de la localité de Bafoussam au Cameroun s'est fait à cause de l'absence des données épidémiologiques récentes en terme de prévalence des virus de l'hépatite B, de l'hépatite C et du VIH de cette localité. Les unités mobiles de dépistage étaient installées entre le marché B de Bafoussam et l'hôpital régional de ladite localité. Les patients dépistés positifs étaient référés au centre de traitement agrée pour le VIH et les hépatites virales de l'hôpital régional de Bafoussam. Nous avons obtenu le consentement éclairé de tous les participants à l’étude pour le dépistage des virus des hépatites B et C [12] et pour le VIH [13–15] ainsi qu'une clairance administrative et éthique. Les pre-counseling et post-counseling ont été effectués par le personnel qualifié des unités mobiles de dépistage [12, 13]. Les données collectées ont été saisies et analysées par des méthodes de statistiques descriptives et analytiques en utilisant le logiciel SPSS 19. Les différences entre les proportions ont été analysées en utilisant des tables de contingence et en appliquant le test de Chi-2. L'ampleur de l'association entre deux variables qualitatives a été évaluée par le rapport de côte pour déterminer les facteurs de risque pris individuellement (analyse univariée). Le test de Cochran-Armitage [16, 17] a été utilisé pour déterminer l'existence d'une variation linéaire de proportions en fonction d'une variable ordinale. Les valeurs p<0,05 étaient considérées comme statistiquement significatives.

## Résultats

La population étudiée était constituée de 982 personnes dont 429 personnes de sexe masculin (43,7%) et 553 de sexe féminin (56,3%) (Tableau 1). La tranche d’âge la plus représentée était celle des 20 à 24 ans. L’âge médian était de 34,5 ans. La population était constituée majoritairement de personnes mariées. La prévalence du VIH était de 6%. Cette prévalence était 2 fois plus élevée chez les femmes que chez les hommes (8,1% contre 3,5%) (p=0,01) (Tableau 2). Les prévalences les plus élevées ont été observées chez les personnes de 30 à 34 ans et de 40 à 44 ans avec 15,0% et 11,5% (p=0,01), les personnes sans emploi (11,1%) (p<0,001) et les personnes en union libre (17,9%) (p=0,000). La prévalence du VIH n’était pas liée aux comportements et pratiques sexuels de la population de l’étude (Tableau 3, Tableau 4). On enregistrait une prévalence très élevée du VIH (29,3%) chez les individus ayant déclaré avoir au moins une infection sexuellement transmissible (p=0,000) (Tableau 5). Les prévalences de l'antigène de surface du virus de l'hépatite B (AgHBs) et de l'anticorps du virus de l'hépatite C (anti HCV) étaient respectivement de 4,1% et de 0.4%. La distribution de ces prévalences en fonction de certaines caractéristiques socio-démographiques fait l'objet des Tableau 6 et Tableau 7.

**Tableau 1:** Répartition de la population par tranche d’âge

Tranche d’âge	Homme	Femme	Ensemble	Pourcentage (%)
15-19	20	46	66	6,7
20-24	69	106	175	17,8
25-29	64	86	150	15,3
30-34	38	62	100	10,2
35-39	53	61	114	11,6
40-44	47	77	124	12,6
45-49	43	39	82	8,4
50 et plus	95	76	171	17,4
Total	429	553	982	100,0

**Tableau 2:** Prévalence du VIH en fonction des caractéristiques sociodémographiques

Variables	Effectif (982)	Prévalence n(%)	RC	95%IC	Valeur p (RC)	Valeur p (Chi-deux)
**Sexe**						
Femme	553	45(8,1)	2,44	(1,34; 4,45)	0,00	0,003
Homme*	429	15(3,5)	1			
**Age (ans)**						
15-19	66	0(0)	NA	NA	NA	NA
20-24	175	4(2,3)	0,43	(0,13; 1,43)	0,08	0,000
25-29	150	4(2,7)	0,51	(0,15; 168)	0,13	
30-34	100	15(15,0)	3,25	(1,37; 7,74)	0,00	
35-39	114	12(10,5)	2,17	(0,88; 5,33)	0,05	
40-44	124	14(11,5)	2,35	(0,98; 5,61)	0,03	
45-49	82	2(2,4)	0,46	(0,10; 2,18)	0,16	
≥50*	175	9(5,3)	1			
**Statut matrimonial**						
Marié(e)	467	25(5,4)	0,26	(0,12; 0,55)	0,00	0,000
Célibataire	385	17(4,4)	0,21	(0,10; 0,47)	0,00	
Veuf (ve)	53	6(11,3)	0,59	(0,20; 1,68)	0,15	
Union libre*	67	12(17,9)	1			
Divorcé(e)	10	0(0)	NA	NA	NA	NA
**Profession**						
Privé	344	24(7,0)	0,60	(0,33; 1,10)	0,05	0,003
Fonctionnaire	205	12(5,9)	0,50	(0,24; 1,03)	0,03	
Elève	128	3(2,3)	0,19	(0,06; 0,65)	0,00	
Sans emploi*	198	22(11,1)	1			
Etudiant	105	0(0)	NA	NA	NA	NA
Retraite	2	0(0)	NA	NA	NA	NA

RC=Rapport de Cote

* = Niveau de Reference pour RC

95%IC=Intervalle de confiance à 95% NA=Non applicable; n=Effectif

**Tableau 3:** Prévalence (%) du VIH et comportements et pratiques sexuels par sexe

Caractéristique du comportement sexuel	Hommes		Femmes		Ensemble	
	n (prévalence)	Effectif	n (prévalence)	Effectif	n (prévalence)	Effectif
**Âge au premier rapport sexuel**						
Moins de 15	5 (7,9)	63	7 (11,3)	62	12 (9,6)	125
15 – 18	1 (1,1)	93	13 (9,6)	135	14 (6,1)	228
18 – 20	7 (4,0)	175	17 (7,1)	241	24 (5,8)	416
20 ou plus	2 (2,7)	75	6 (9,2)	65	8 (5,7)	140
**Valeur-P**	0,155		0,676		0,465	
**Nombre de partenaires sexuels au cours des 12 derniers mois**						
0	0 (0,0)	6	2 (6,5)	31	2 (5,4)	37
1	6 (3,5)	172	32 (8,5)	376	38 (6,9)	548
2	4 (3,8)	104	5 (7,8)	64	9 (5,4)	168
3	2 (2,7)	75	3 (13,6)	22	5 (5,1)	97
4 ou plus	3 (6,1)	49	1 (10,0)	10	4 (6,8)	59
**Valeur-P**	0,863		0,914		0,925	
**Nombre de partenaire occasionnel**						
0	10 (4,1)	343	32 (8,7)	368	42 (6,9)	569
1	3 (3,8)	80	8 (7,7)	104	11 (6,0)	173
2	1 (2,6)	38	1 (4,8)	21	2 (3,4)	57
3	1 (4,2)	24	1 (20,0)	4	2 (6,9)	27
4 ou plus	0 (0,0)	21	1 (20,0)	4	1 (3,8)	25
**Valeur-P**	0,9		0,704		0,831	
**Utilisation du préservatif**						
Utilise toujours	3 (1,9)	161	24 (15,1)	159	27 (8,4)	293
Utilise quelques fois	11 (5,4)	202	16 (5,8)	278	27 (5,6)	453
Jamais utilisé	1 (2,3)	43	3 (4,5)	66	4 (3,7)	105
**Valeur-P**	0,175		0,002		0,131	
**Utilisation d'un préservatif lors du dernier rapport sexuel avec une prostitué**						
Oui	2 (4,0)	50	3 (21,4)	14	5 (7,8)	64
Non	0 (0,0)	2	0 (0,0)	2	0 (0,0)	4
**Valeur-P**	0,956		0,191		0,753	

**Tableau 4:** Prévalence (%) du VIH, nombre de partenaires et type de rapport sexuels par sexe

Caractéristique du comportement sexuel	Hommes		Femmes		Femmes	
	n(prévalence)	Effectif	n(prévalence)	Effectif	n(prévalence)	Effectif
**Nombre de partenaire sexuel tout au long de la vie**						
0	0 (0,0)	1	0 (0,0)	1	0 (0,0)	2
1	2 (8,7)	23	5 (4,8)	104	7 (5,5)	127
2	0 (0,0)	47	9 (7,6)	119	9 (5,4)	166
**Nombre de partenaire sexuel tout au long de la vie**						
0	0 (0,0)	1	0 (0,0)	1	0 (0,0)	2
1	2 (8,7)	23	5 (4,8)	104	7 (5,5)	127
2	0 (0,0)	47	9 (7,6)	119	9 (5,4)	166
3 à 4	2 (2,0)	101	5 (3,4)	147	7 (2,8)	248
5 à 9	9 (5,0)	180	20 (16,9)	118	29(9,7)	298
10 ou plus	2 (3,7)	54	4 (28,6)	14	6 (8,8)	68
**Valeur-P**	0,397		0,000		0,034	
**Rapport sexuel anal**						
Une fois	1 (2,3)	42	4 (11,4)	35	5 (6,4)	77
Régulièrement	0 (0,0)	63	3 (4,3)	69	3 (2,3)	132
Jamais	14 (4,7)	286	36 (9,0)	399	50 (7,2)	685
**Valeur-P**	0,179		0,36		0,109	
**Rapport sexuel en groupe**						
Oui	0 (0,0)	12	3 (15,8)	19	3 (9,7)	31
Non	15 (3,8)	394	40 (8,3)	484	55 (6,3)	878
**Valeur-P**	0,491		0,250		0,445	

**Tableau 5:** Prévalence (%) du VIH en fonction des antécédents d'IST

IST	Hommes		Femmes		Ensemble	
	n (prévalence)	Effectif	n (prévalence)	Effectif	n (prévalence)	Effectif
Oui	7 (13,0)	54	17 (29,3)	58	24 (21,4)	112
Non	8 (2,3)	352	26 (5,8)	445	34 (4,3)	870
Valeur-P	0,000		0,000		0,000	

**Tableau 6:** Prévalence de l'AgHBs selon les caractéristiques sociodémographiques

Caractéristiques	Effectif (982)	Prévalence n(%)	RC	95%IC	Valeur-P (RC)	Valeur-P (Chi-deux)
**Sexe**						
Femme	553	20(4,7)	0,73	(0,40; 1,36)	0,16	0,502
Homme*	429	21(3,8)	1			
**Age**						
15-19	66	4(6,1)	0,40	(0,12; 1,26)	0,06	0,000
20-24	175	5(2,9)	0,18	(0,06; 0,52)	0,00	
25-29	150	9(6,0)	0,39	(0,16; 0,94)	0,02	
30-34*	100	14(14,0)	1			
35-39	114	5(4,4)	0,28	(0,10; 0,81)	0,00	
40-44	124	0(0,0)	NA	NA	NA	
45-49	82	3(3,7)	0,23	(0,06; 0,84)	0,01	
≥50	175	1(0,6)	0,03	(0,01; 0,27)	0,00	
**Statut matrimonial**						
Marié(e)	467	13(2,8)	0,48	(0,13; 1,73)	0,13	0,265
Célibataire	385	21(5,5)	0,96	(0,28; 3,34)	0,47	
Union libre	67	3(4,5)	0,78	(0,15; 4,04)	0,38	
Divorcé(e)	10	1(11,1)	1,85	(0,17; 19,85)	0,30	
Veuf (ve)*	53	3(5,7)	1			
**Profession**						
Privé	344	21(6,1)	1,64	(0,55; 4,89)	0,19	0,427
Fonctionnaire	205	7(3,4)	0,89	(0,26; 3,12)	0,43	
Elève	128	5(3,9)	1,03	(0,27; 3,92)	0,48	
Sans emploi	198	4(2,0)	0,52	(0,13; 2,13)	0,18	
Etudiant*	105	4(3,8)	1	NA	NA	
Retraite	2	0(0,0)	NA	NA	NA	

RC=Rapport de Cote

* = Niveau de Reference pour RC

95%IC=Intervalle de confiance à 95% NA=Non applicable; n=Effectif

**Tableau 7:** Prévalence de l'hépatite C en fonction de l’âge et du sexe

Variables	Fréquence	Prévalence n(%)	P-Value
**Sexe**			
hommes	429	2(0,5)	0,502
femmes	553	2(0,4)	
**Age (ans)**			
15-24	241	0(0)	0,000
25-34	250	1(0,4)	
35-44	238	1(0,5)	
45 et plus	253	2(0,7)	
**Total**	982	4(0,4)	

## Discussion

Ce travail avait pour but d’étudier la séroprévalence et les facteurs associés au VIH et aux hépatites virales B et C dans la ville de Bafoussam au Cameroun. Il s'agissait d'une étude descriptive et analytique menée de Février à Juin 2012. Nous avons recruté 982 participants après avoir obtenu leur consentement éclairé. Ces derniers ont bénéficié chacun d'un dépistage gratuit du VIH, de l'Antigène HBs du virus de l'hépatite B et de l'Anticorps anti HCV du virus de l'hépatite C. Les sérologies étaient réalisées le jour même des prélèvements afin d'optimiser le retrait des résultats. La majorité des personnes dépistées étaient les femmes (56,3%). Ce chiffre est inférieur à celui retrouvé par Mbopi-Kéou et collaborateurs [14] où 64,5% des volontaires ayant participé aux campagnes de sensibilisation sur une période de trois ans étaient des femmes. Dans une société se modernisant davantage, les femmes s'assumeraient plus et seraient plus enclines à se protéger donc se faire dépister. L’âge moyen de la population était 34,5 ans. Ce qui dénote une considération accrue des jeunes de cette tranche d’âge pour les mesures préventives contre le VIH/SIDA et les infections associées.

La prévalence de l'infection par le VIH était de 6,0%. Ce qui est supérieure à la prévalence au Cameroun qui est de 4,3% selon l'enquête de démographie et de santé (EDS-MICS 2011) [18]. Ceci serait peut-être aussi lié au fait que certains séropositifs confirmés se présentaient afin de pouvoir bénéficier gratuitement des tests de dépistage pour les virus des hépatites virales B et C. La prévalence de l'infection par le VIH chez les femmes était deux fois plus élevée que chez les hommes. Ce qui montre la susceptibilité de la femme à l'infection par le VIH probablement liée aux prédispositions anatomiques. Cette observation suit la tendance de la féminisation de l'infection décrite par l'EDS III 2010 (6,8% contre 4,1%) [19]; par l'EDS-MICS 2011 (5,6% contre 2,9%) [18]; ainsi que par Mbopi-Kéou et collaborateurs qui avaient observés une évolution de la prévalence au sein de la population féminine allant de 6,7% en 2005 à 9,73% en 2008 [20]. Les prévalences les plus élevées étaient observées après 30 ans. Cette observation corrobore celle de Mbopi-Keou et collaborateurs dans le cadre d'une campagne de santé à Yaoundé au Cameroun, où le pic de fréquence était atteint après 40 ans [21]. Cette prévalence de l'infection par le VIH était plus élevée parmi les personnes en union libre (17,9%) soit 4 fois plus élevée que chez les célibataires. L'union libre constituerait donc un facteur de risque probablement lié à la notion d'engagement partiel qui laisserait libre cours aux comportements sexuels à risque. Les sans emplois représentaient la classe la plus atteinte avec 11,1%. Cette prévalence se rapproche de celle d'une étude antérieure par Mbopi-Kéou et collaborateurs à Yaoundé où cette catégorie représentait 10,6% [21]. La prévalence du VIH n’était pas directement liée aux comportements et pratiques sexuels de la population de l’étude. Cette observation corrobore celle de Mossoko et collaborateurs au Cameroun [22] où l’âge du premier rapport n'influençait pas la prévalence du VIH.

Les infections sexuellement transmissibles (IST) jouent un rôle dans la transmission sexuelle du VIH [23, 24]. La prévalence de l'infection par le VIH était plus élevée chez les personnes ayant déclaré avoir eu une IST ou un symptôme d'IST. Cette observation corrobore celles de Mbopi-Keou et collaborateurs et de Mossoko et collaborateurs [22–24] La prévalence du virus de l'hépatite B (VHB) dans notre étude était de 4,1%. Elle était inférieure à celle enregistrée dans une étude précédente au Cameroun où elle était de l'ordre de 10% [25]. Ceci peut s'expliquer par le fait que distribution du VHB dans les différentes régions du Cameroun n'est pas uniforme. Quant au virus de l'hépatite C (VHC), sa prévalence était de 0,4%, bien en déça de l’étude de Nerrienet et collaborateurs qui rapportaient une prévalence de 13% [26]. Cette différence montre la disparité dans la répartition de l'infection par le VHC allant de 0,6% chez les pygmées [25] à 16,9% au sud du Cameroun [26].

## Conclusion

Cette étude démontre à suffire l'urgence de mettre en place des stratégies de prévention contre le VIH, les hépatites virales et les facteurs associés au Cameroun.
